# Regional Anesthesia in Children: How Do We Know It Works? A Review of a Novel Tool for Assessing the Impact of Regional Anesthesia for Pediatric Surgical Patients

**DOI:** 10.3390/children12091117

**Published:** 2025-08-25

**Authors:** David L. Moore, Lili Ding, Fang Yang, Jiwon Lee, Senthilkumar Sadhasivam, Ali Kandil

**Affiliations:** 1 Department of Anesthesiology and Pediatrics, Cincinnati Children’s Hospital Medical Center, Cincinnati, OH 45229, USA; ali.kandil@cchmc.org; 2 Division of Biostatistics and Epidemiology, Cincinnati Children’s Hospital Medical Center, Cincinnati, OH 45229, USA; lili.ding@cchmc.org (L.D.); yangf8@mail.uc.edu (F.Y.); lee9j2@mail.uc.edu (J.L.); 3 Department of Anesthesiology and Perioperative Medicine, University of Pittsburgh Medical Center, Pittsburgh, PA 15213, USA; sadhasivams@upmc.edu

**Keywords:** regional anesthesia, opioid sparing, epidural analgesia, pediatric surgical pain

## Abstract

**Highlights:**

**What are the main findings?**

- Over the night of surgery (NOS), the regional anesthesia (RA) group consumed a significantly lower amount of opioids compared with that in the non-RA group (median morphine equivalency rate (MER) = 4.06 mcg/kg/h versus 16.16 mcg/kg/h; *p* < 0.001).
- A proportion of >25% (Lower Quartile: IQR) of our patient’s MERs were zero (needing no IV opioids) for the NOS, POD#1, and POD#2 in the regional and epidural cohorts, despite all having reasonable access to IV opioids.

**What is the implication of the main finding?**

- Many patients did not need IV opioids for satisfactory pain relief, indicating a successful block.
- Despite particularly rigid pain protocols, the variation in pain scores and IV opioids demonstrates that despite patients being treated the same, there is individual variation at play in postoperative pain control.

**Abstract:**

Objectives: We wished to demonstrate the utility of a novel quantitative assessment tool for the efficacy of regional anesthesia in children. Methods: The authors examined the records of all patients evaluated by the acute pain service during a 6-month period at a large quaternary-care pediatric hospital. The morphine equivalency rate (MER) in mcg/kg/hour was employed to compare the opioid use in children undergoing similar procedures with and without regional anesthesia (RA). Results: A total of 744 patients were included in this study, 333 of whom received RA. The RA group demonstrated a statistical and clinical benefit from having regional anesthesia, as demonstrated by the MER, compared to the non-RA group. Discussion: Objective measurements of RA in adults are overshadowed by subjective evidence of surgical tolerance in awake or lightly sedated patients. However, in pediatrics, objective measures are still needed to highlight the importance and utility of RA. Such objective tools could impact the adoption of RA by our surgical colleagues and have a long-term impact on opioid use and even abuse. We implemented the MER to quantify the benefit of RA. Given the adverse effects of opioids on gut motility, the incidence of nausea and vomiting, hypersensitivity reactions, and ubiquitous potential for abuse, the MER objectively demonstrates RA’s impact on pediatric surgical patients and why its utilization as an assessment tool could ultimately change practice.

## 1. Introduction

Evaluating the impact of regional anesthesia (RA) in pediatric patients is no easy task. In adults, RA may serve as the sole anesthetic, and completion of the surgical procedure in a still patient is evidence enough for the success of the block. Unfortunately, cases of patients being awake under RA are rare in pediatric surgical procedures. Therefore, another endpoint for gauging block success is needed.

In a pilot study we conducted using a target device designed to gauge ultrasound skills, we found that high speed and accuracy of hitting a target using the ultrasound closely mimicked expert behavior [1,2,3]. As a corollary to these studies, we implemented a new procedural assessment of pediatric regional anesthesia that focused on “performance times” and “procedural success”. We recorded the “performance times” easily. Despite having motor block and sensory block endpoints, we tended to grade “procedural success” more subjectively. The efficacy of analgesics appeared to be poorly related to these subjective outcomes [4,5].

Initially, we considered assessing the efficacy of preoperative regional anesthesia through the amount of intraoperative medications (volatile anesthetics and narcotics) needed, as these should be diminished with a successful block [5,6,7,8,9,10]. Unfortunately, these other anesthetics complicate an intraoperative assessment of the success of regional anesthesia, making the isolation of regional effects overly complex and cumbersome. Also, we perform many of our blocks at the end of a case, rendering intraoperative evaluations moot. Evaluating the success of regional anesthesia during the postoperative period diminishes the confounding factor of polypharmacy [11]. The major contributors to analgesia during this time are opioids and regional anesthesia [8,12]. When comparing the opioid usage among variably sized patients (e.g., premature infants through morbidly obese adults), standardization can be achieved by using the morphine equivalency rate (MER), which includes weight-based rates with only intravenous opioids converted into morphine.

Anesthesia providers administer opioids as one of the primary drugs for postoperative analgesia. Opioids continue to be the pinnacle of the World Health Organization Pain pyramid [13]. Unfortunately, opioids also negatively affect patients, thereby impacting postoperative care and course by contributing to excessive sedation, postoperative nausea and vomiting (PONV), ileus, and constipation—increasing the length of hospital stays [5,6,7,8,9]. They also impair ventilation in a dose-dependent manner, but fortunately, regional anesthesia may decrease the risk of respiratory depression through its opioid-sparing effect [10].

Regional anesthesia may decrease or eliminate the need for intravenous (IV) opioids [6,14,15]; improve static and dynamic pain control; decrease PONV; and minimize the risks of chronic postsurgical pain [12,16,17,18]. McBride et al. noted the benefits of regional anesthesia when analyzing epidural analgesia in pectus excavatum repair surgeries [19]. Unfortunately, some clinicians remain skeptical of the risk–benefit ratio of regional anesthesia, where some studies have demonstrated no differences in patient satisfaction, nausea, emesis, or pruritus [20,21,22].

We aim to validate a novel regional anesthesia assessment tool that quantitatively measures its impact on pediatric perioperative pain management. Using a retrospective analysis of the postoperative IV opioid consumption in a large pediatric surgical cohort, we demonstrate the tool’s potential to inform practice. By replacing subjective assessments with objective metrics such as the morphine equivalency rate (MER), we strive to support the broader adoption of regional anesthesia and advance evidence-based pain management.

## 2. Materials and Methods

Study design: We conducted a retrospective review of the Epic electronic medical records (EMRs) of all patients followed by the acute pain service (APS) at Cincinnati Children’s Hospital Medical Center over a 6-month period. The institutional review board (IRB) at Cincinnati Children’s Hospital Medical Center reviewed and approved this study. We looked at the clinical IV opioid usage through patient-controlled analgesia (PCA) investigations and review of the EMRs.

Tracking opioid usage: We totaled the IV opioid use for discrete times over the perioperative period for all patients as follows: (1) Intraoperative time included “anesthesia start” until “anesthesia end”, thus accounting for all medications given by the intraoperative anesthesia provider. (2) Night of surgery (NOS) included the time between “anesthesia end” and 7 AM on postoperative day (POD)#1. We started most of the PCAs in the post-anesthesia care unit (PACU), and this accounted for all opioid use in the PACU and on the ward until nursing cleared the PCA totals, typically by 6 AM the next day. The NOS time varied due to the random times at which surgeries ended; therefore, the hours were not uniform. To overcome this limitation, we tracked rates (the opioid use per hour) rather than only tracking 24 h periods. (3) “POD #1” started at 7 AM on POD#1 and ended at 7 AM on POD #2. (4) “POD #2” included IV opioids from 7 AM on POD#2 through to 7 AM on POD#3. We completed most assessments of the blocks by assessing the intraoperative and NOS timepoints, and there appeared little need to follow up past POD#2. These time intervals most accurately coincided with acute postoperative pain and reflected a natural cohort.

Morphine equivalency rate (MER): We recorded the amounts of perioperative IV opioids used and then converted this amount into MER (micrograms of morphine used/patient weight in kilograms/period of time assessed in hours) using established opioid conversions [23]. The conversion factors were 50 mcg of fentanyl to 1 mg of morphine and 0.2 mg of hydromorphone to 1 mg of morphine. The amounts were additive between different opioids. Once converted and totaled to micrograms of morphine, we divided the opioids by the patient’s weight in kilograms and divided again by the period of time assessed in hours. This is a problem with previously published articles on opioid sparing, as the opioid sparing is reported in total amounts or percentages [8,11]. The MER was used to assess the success of regional anesthesia by comparing it with established norms, such as a “typical” postoperative morphine infusion rate of 20 mcg/kg/h. A successful block needs little to no rescue (as in a typical C-section). Therefore, the lower the MER, the more successful the block.

Statistical analysis: We generated descriptive statistics for all cohorts, as the frequency and percentage for categorical variables and as the mean, standard deviation, median, and interquartile range (IQR) for continuous variables. From the EMR, we extracted and analyzed the opioid totals, weight, age, time, gender, type of surgical procedure, surgical duration, and method of regional anesthetic.

**Figure 1:**
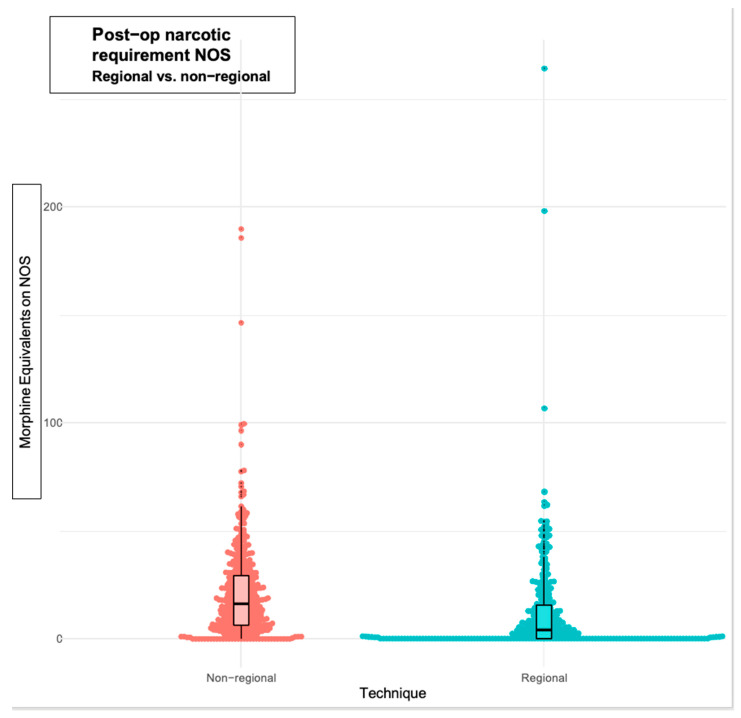
Bee swarm comparison.

We accepted statistical significance at *p* < 0.05. The Kolmogorov–Smirnov and Shapiro–Wilk tests determined the normality of the dataset. We assessed age and weight using the two-sample *t*-test, as they were normally distributed. As the operating room time and the MER groups were non-parametric, we compared the datasets by using Wilcoxon scores (rank sums). We assessed each patient for their individual MER and then grouped them by similarity, comparing the groups by median MER. Initially, we compared the median MER of “Regional” cases against the median MER of all “Non-Regional” cases. This was considered to be a gross comparison due to the various amounts of pain and narcotic requirements for various surgeries. Subsequent group comparisons used increasingly more specific cohorts, such as all “Thoracic Regional” cases compared to all “Thoracic Non-Regional” cases or “Single-shot Transversus Abdominis Plane block” cases versus all “Abdominal Non-Regional” cases. Finally, we formed groups based on the CPT surgical codes and assessed each group’s median and interquartile range. We used a bee swarm comparison to visualize each group (Figure 1).

In this bee swarm comparison, each individual patient is plotted by their MER for the NOS and divided by the use of non-regional or regional techniques. When comparing all individual non-regional versus all individual regional cases, it is helpful to understand the value of zeroes (no IV opioids). In the figure, the regional side shows much greater values at zero and near-zero, as seen using both the bee swarm data technique and the box and whisker plot showing the median value along with the interquartile range. The units for the MER (y-axis) are mcg morphine/kg/h.

Power analysis: We conducted a power analysis for the primary outcome of the MER on the NOS comparing between the overall regional and non-regional groups. The power analysis was based on 2000 Monte Carlo samples from the null and alternative distributions (gamma) of the outcome estimated using the data in the two groups, where null values (zeros) were shifted by a small positive number. Under the null hypothesis, the two groups have the same gamma distribution. Group sample sizes of 411 and 333 achieve more than 99% power for rejecting the null hypothesis using a two-sided Wilcoxon rank sum test. The significance level (alpha) was set at 0.0125, and Bonferroni multiple comparison correction was used for the four primary comparisons (the MER at the NOS for overall regional vs. non-regional; thoracic regional vs. non-regional; abdominal regional vs. non-regional; and abdominal epidural vs. non-regional).

## 3. Results

Overall, we evaluated 744 patient records. Our participants included every patient followed by the acute pain service (APS) over a 6-month period at Cincinnati Children’s Hospital Medical Center. Patients (including many day surgery patients with caudal blocks) not followed by the APS will have been the only group excluded from this study. The mean age was 11.46 years, with an SD of 7.64. The minimum age was 0.17 years, and the maximum was 47 years. Of the 411 in whom RA was not used, 216 were female, and 195 were male. Of those using RA, 155 were female, and 178 were male (Table 1).

Over the night of surgery (NOS), the RA group consumed a significantly lower amount of opioids compared with that in the non-RA group (median MER = 4.06 mcg/kg/h versus 16.16 mcg/kg/h; *p* < 0.001). Thus, the children receiving blocks required only approximately one-quarter of the IV opioids that the non-RA children did [24]. We also compared smaller subsets of patients, including regional abdominal cases (147 patients) and non-regional abdominal cases (176 patients). The results of this comparison mirrored those for the entire cohort. The regional abdominal group consumed a significantly lower amount of opioids compared with that in the non-regional abdominal group (median NOS MER of 5.22 mcg/kg/h versus 14.30 mcg/kg/h; *p* < 0.001). We also examined all thoracic cases. The regional thoracic group (40 patients) utilized a median NOS MER of 6.56 mcg/kg/h compared with 18.72 mcg/kg/h for non-regional thoracic cases (42 patients; *p* < 0.01). Patients receiving epidural abdominal analgesia compared favorably with the non-regional abdominal cases. Epidural analgesia was almost completely opioid-sparing, as the median MER over the NOS was 0.78 mcg/kg/h compared with 14.30 mcg/kg/h for the non-regional cases (*p* < 0.001). In the far-right column, the numbers comprise the percent of patients that required no IV opioids over the NOS. Of note, almost half of the patient that received an abdominal epidural did not require any IV opioids on the NOS (Table 2).

**Figure 2:**
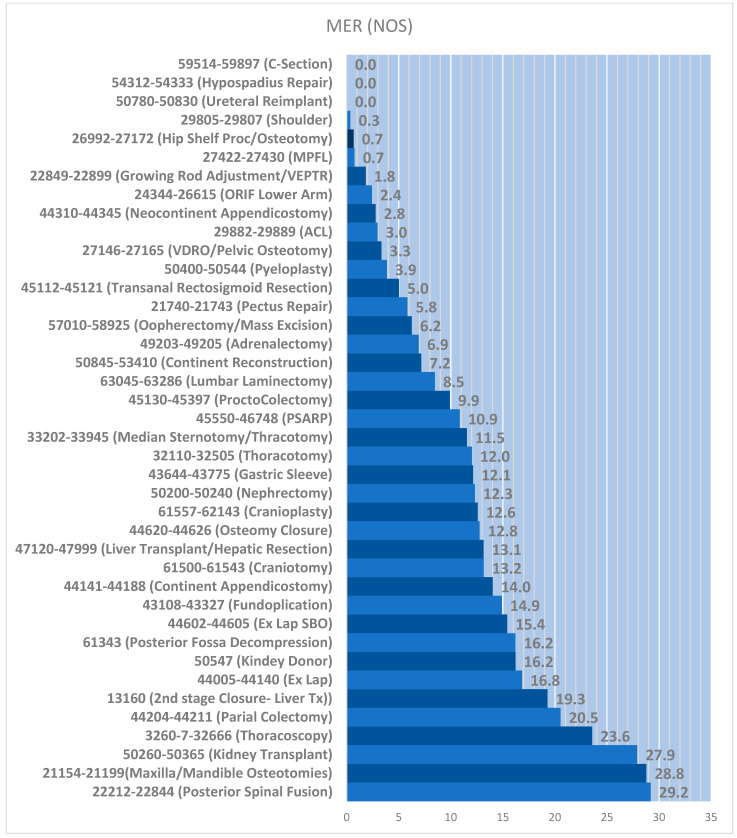
Subgroup stratification for NOS using CPT codes.

Finally, we compared the patients according to surgical CPT codes [25]. The figure lists the CPT-specific surgeries, organizing the cases by the MER, graphing their MERs for the night of surgery (NOS) (Figure 2). The table following the figure lists these same CPT-specific surgeries, with the case numbers, NOS MERs, and the types of regional blocks used in these cases (Table 3).

The first column lists the specific CPT codes and procedures. The median MER is noted to the right of each bar. The units for the MER (x-axis) are mcg morphine/kg/h.

## 4. Discussion

Determining the success of RA invites subjectivity. The evaluator may consider it successful merely upon the placement alone [4,26]. The physician evaluating it postoperatively may consider it successful based upon the overall comfort of the patient or upon pain scores [27,28]. Another person might call it successful based on sensory levels or motor blockade [29]. In this study, we confirmed the postoperative opioid-sparing effect of RA and were able to quantify impact and success of the block using the MER.

One assumption that is necessary for the utilization of the MER is that when adequate analgesia is administered, patients will be satisfied. Similarly, we previously described that a low MER secondary to an inadequate and purposeful opioid-“sparing” plan correlated with dissatisfaction [30]. McQuay et al. noted in 2012 when comparing different pain modalities, “Unsurprisingly, patient satisfaction is highly correlated with good pain relief…” [31], providing an update from an article from 2008 were he conjectured “the analgesic consumption outcome measure is valid only when treatment groups achieve similar pain scores” [32]. Gerbershagen et al. in 2013 demonstrated that pain scores did not appear to be correlated with the amount of tissue trauma but rather the arbitrariness of the pain plan [28]. Their study noted many “minor surgeries” with high pain scores “…because these patients are given less analgesia than needed”, while “comparatively low pain scores were observed after a number of major abdominal surgeries, often due to sufficient epidural analgesia” [28].

We utilized the MER as a tool to evaluate postoperative pain for several reasons. First, the MER grossly demonstrated the success of the block by lower opioid usage for regional cases compared with that in non-regional cases (Table 2). These results were similar to those from other studies [33,34,35,36]. Numerically assessing the analgesic efficacy of a block is possible and relatively easy. Many patients did not need IV opioids for satisfactory pain relief, indicating a successful block. This was so common that >25% (Lower Quartile: IQR) of our patient’s MERs were zero (meaning they needed no IV opioids) for NOS, POD#1, and POD#2 in the regional and epidural cohorts (Table 2), despite all having reasonable access to IV opioids. This was true also during POD#1 and POD#2 in the thoracic regional and single-shot transversus abdominus plane (SSTAP) cohorts. This confirms a well-known advantage of regional anesthesia when compared to similar cases without regional anesthesia [28]. Minimizing IV opioid rescue helped to account for our non-parametric results in all cohorts.

Second, the MER helped determine whether the block was accurately placed under anesthesia. We composed this metric in two parts, as described previously [2]. We noted speed by time of placement and success by the relative MER. From our data in a teaching hospital, single-shot blocks took ~5 min and catheters took ~15 min on average. Best practice would comprise faster placement with a low MER. The MER objectively describes these gradations, stratifying all surgeries into the amount of IV opioid use relative to that for other surgeries. So, anesthesia can note whether the block’s MER compares favorably relative to the cohort’s median MER or even against that for the standard of a routine morphine infusion (20 mcg/kg/h) (Table 2) (Figure 2).

Third, we used the MER to assess how well a patient tolerated pain. All pain plans can use the MER to assess variation. This is even true with non-regional cases. Despite particularly rigid pain protocols, we still see large variations in the pain scores and tolerance of these surgeries. This means that despite patients being treated the same, there is individual variation at play in postoperative pain control. We saw high MERs in chronic pain patients, as well as patients with block problems (one-sided blocks, block placement misaligned with the incision, a low volume of local anesthetic). Interestingly, many of the patients with the highest MERs also had severe anxiety/agitation. For these anxious/agitated patients, when treated with anxiolytics, their MERs tended to normalize towards the median.

When we evaluated our results comparing subgroups of regional anesthesia, neuraxial cases showed the most reduced MERs, demonstrating the superior analgesic efficacy of neuraxial analgesia in children compared to that of other analgesic strategies. Continuous epidural analgesia offered the best analgesia in children for major inpatient surgery, and single-shot caudal analgesia offered the best analgesia for outpatient surgeries in children. Comparing the median values (2.17 for epidural, 16.2 for non-regional) (*p* < 0.001) again clearly showed significant differences between the groups. We believe that this objective assessment of the success of regional anesthesia could have favorable effects on the adoption of regional anesthesia by different institutions and our surgical colleagues. Such increased adoption could contribute to opioid-sparing efforts.

The strength of this study is that it is the first large study in children objectively quantifying the opioid-sparing effects of regional anesthesia. It was a quality improvement pilot study that quickly outgrew its original intent to become a database. We therefore did not exclude anyone from the care of the acute pain service over that 6-month period. Limitations of this study were the retrospective, observational, single-site design and the lack of assessment of the pain scores. Additionally, subdividing the groups based on the surgical procedure reduced the sample size but provided a procedure-specific and objective comparison of regional and non-regional analgesic efficacy.

## 5. Conclusions

We demonstrated the utility of quantifying postoperative opioid usage and opioid sparing. We can use the MER objectively as a metric to assess the analgesic efficacy of regional anesthesia in children. Properly performed regional anesthesia will objectively decrease postoperative opioid requirements in terms of the MER. Again, a quantitative assessment tool that demonstrates such a reduction in opioid requirements could help minimize dependence on opioids by increasing the adoption of regional anesthesia by other institutions and our surgical colleagues. Further, this quantification allows for an objective assessment of block success, skill assessments for different regional practitioners, scrutiny of outliers, and the stratification of surgery-specific pain, guiding the optimal selection of postoperative analgesia for specific pediatric surgical procedures.

## Figures and Tables

**Table 1:** Demographic data.

Regional Blocks ^a^								
No			411 (55.24%)					
Yes			333 (44.76%)					
Gender ^a^								
Female			371 (49.87%)					
Male			373 (50.13%)					
	N	Mean	Std. Dev		Min		Maximum	
Age ^b^ (yr)	743	11.46	7.64		0.17		47	
Weight ^b^ (kg)	744	44.71	29.96		4.47		202.2	
	N	Min	Q1	Median		Q3		Maximum
OR Time ^c^ (h)	743	0.72	3.13	4.35		6.22		19.15
MER Interop ^c^	741	0	18.52	32.54		49.89		461.17
MER NOS ^c^	741	0	2.1	10.3		24.92		264.15
MER POD#1 ^c^	742	0	0	2.84		17.13		191.2
MER POD#2 ^c^	742	0	0	0		5.52		191.74

^a^ For both regional blocks and gender, frequency and percentage are utilized. ^b^ Age and weight—both parametric—mean ± SD used. ^c^ OR time and MER—non-parametric—median (IQR) used. MER = morphine equivalency rate (mcg morphine/kg/h).

**Table 2:** Cohort comparisons of the MER for patients with and without a regional block stratified by type of surgery.

	Number	MER (OR) ^a^	MER NOS ^b^	MER = 0 on NOS ^c^ (% of pts)
	#	Median (IQR)	Median (IQR)	
Non-Regional	411	39.5 (21.9–61.8)	16.2 (6.2–29.2)	8.1
All Regional	333	26.2 (13.9–38.2)	4.1 (0–15.6)	32.3
Non-Regional Thoracic	42	68.0 (37.6–94.2)	18.7 (6.8–38.5)	11.9
Regional Thoracic	40	22.6 (14.8–35.6)	6.6 (3.2–16.46)	7.5
Non-Regional Abdominal	176	47.0 (33.6–70.3)	14.3 (6.2–28.6)	7.4
Regional Abdominal	147	27.2 (14.9–38.3)	5.2 (0–16.2)	30.3
Abdominal Epidural	69	18.5 (12.0–26.6)	0.8 (0–4.5)	47.8
Abdominal Single-Shot TAP	33	40.0 (33.9–53.5)	16.0 (8.5–26.7)	6.1
Abdominal TAP Catheter	31	35.8 (30.0–42.9)	15.4 (8.9–37.8)	0
Non-Regional Laparoscopy	52	44.4 (38.7–61.6)	12.7 (4.6–29.6)	5.8
Non-Regional Laparotomy	124	47.7 (33.0–74.4)	15.3 (6.2–28.1)	8.1
All Epidurals	156	19.2 (10.4–28.4)	2.5 (0–7.6)	35.9
Caudal ± Duramorph	16	12.8 (8.3–27.0)	0 (0–0)	81.3

^a^ The morphine equivalency rate (MER) in the operating room under anesthesia. ^b^ The morphine equivalence rate (MER) after anesthesia until 7 am on postoperative day 1 (night of surgery). ^c^ Data shown in % of patients on the night of surgery (NOS) that did not use IV opioids (MER = 0). The higher the percent in this column, the more patients avoided all IV opioids, and thus the better the block. All groupings are non-parametric. The median is utilized as the comparator for the groups (with the interquartile range (IQR)). This table also compares all groups, including sample size and all OR and NOS MER medians. The median for the groups is the amount of IV opioids per kilogram of weight per hour used during the case (OR) or after the case (NOS). Units for above columns: MER (mcg morphine/kg/h).

**Table 3:** Subgroup stratification for NOS using CPT codes.

Surgical Codes	Cases	MER (NOS)	Regional %
59514-59897 (C-Section)	5	0	100% Epidural
54312-54333 (Hypospadius Repair)	2	0	100% Caudal%
50780-50830 (Ureteral Reimplant)	40	0	85% Caudal/Epidural; 2.5% TAP
29805-29807 (Shoulder)	14	0.34	100% Intrascalene Block
26992-27172 (Hip Shelf Proc/Osteotomy)	13	0.65	77% Epidural
27422-27430 (MPFL)	29	0.74	100% Femoral; 96.6% Sciatic
22849-22899 (Growing Rod Adjustment/VEPTR)	6	1.82	0%
24344-26615 (ORIF Lower Arm)	9	2.41	44% Infraclav; 22% Supraclav
44310-44345 (Neocontinent Appendicostomy)	5	2.77	20% Epidural; 20% TAP
29882-29889 (ACL)	24	2.96	100% Femoral; 87.5% Sciatic
27146-27165 (VDRO/Pelvic Osteotomy)	34	3.33	85% Epidural
50400-50544 (Pyeloplasty)	8	3.85	75% Epidural
45112-45121 (Transanal Rectosigmoid Resection)	6	5.01	67% Epidural
21740-21743 (Pectus Repair)	32	5.82	97% Epidural
57010-58925 (Oopherectomy/Mass Excision)	9	6.24	11% Epidural; 11% TAP
49203-49205 (Adrenalectomy)	10	6.92	70% Epidural; 30% TAP
50845-53410 (Continent Reconstruction)	9	7.15	33% Epidural/Caudal
63045-63286 (Lumbar Laminectomy)	13	8.46	0%
45130-45397 (ProctoColectomy)	8	9.9	50% TAP
45550-46748 (PSARP)	31	10.85	29% Epidural; 6.5% TAP
33202-33945 (Median Sternotomy/Thracotomy)	27	11.53	0%
32110-32505 (Thoracotomy)	11	12.01	91% Epidural
43644-43775 (Gastric Sleeve)	16	12.13	0%
50200-50240 (Nephrectomy)	7	12.3	28.5% Epidural; 14.2% TAP
61557-62143 (Cranioplasty)	9	12.58	0%
44620-44626 (Osteomy Closure)	25	12.76	8% Epidural; 60% TAP
47120-47999 (Liver Transplant/Hepatic Resection)	13	13.14	7.7% Epidural; 7.7% TAP
61500-61543 (Craniotomy)	15	13.15	0%
44141-44188 (Continent Appendicostomy)	20	14.02	25% TAP
43108-43327 (Fundoplication)	4	14.89	25% Epidural
44602-44605 (Ex Lap SBO)	7	15.41	29% TAP
61343 (Posterior Fossa Decompression)	15	16.2	0%
50547 (Kindey Donor)	13	16.2	100% TAP
44005-44140 (Ex Lap)	19	16.83	10.5% Epidural; 10.5% TAP
13160 (2nd stage Closure- Liver Tx))	8	19.27	0%
44204-44211 (Parial Colectomy)	22	20.53	45% TAP
3260-7-32666 (Thoracoscopy)	14	23.56	7% Epidural
50260-50365 (Kidney Transplant)	20	27.89	15% TAP
21154-21199(Maxilla/Mandible Osteotomies)	8	28.77	0%
22212-22844 (Posterior Spinal Fusion)	71	29.18	0%

The first column lists specific CPT codes and procedures. The next column denotes numbers in groups. The third column is the MER for the NOS. The units for the MER are mcg morphine/kg/h. The final column is the percentage of regional anesthesia types per group.

## Data Availability

The raw data supporting the conclusions of this article can be made available by the authors on request.

## Abbreviations

The following abbreviations are used in this manuscript:

NOS	Night of Surgery
RA	Regional Anesthesia
MER	Morphine Equivalency Rate
IQR	Interquartile Range
IV	Intravenous
mcg	Microgram
kg	Kilogram
h	Hour
POD	Postoperative Day
PONV	Postoperative Nausea and Vomiting
EMR	Electronic Medical Record
APS	Acute Pain Service
IRB	Institutional Review Board
PCA	Patient-Controlled Analgesia
PACU	Post-Anesthesia Care Unit
mg	Milligram
SSTAP	Single-Shot Transversus Abdominis Plane
CPT	Current Procedural Terminology
